# A Case of Hypocalcemia with Severe Vitamin D Deficiency following Treatment for Graves' Disease with Methimazole

**DOI:** 10.1155/2013/512671

**Published:** 2013-04-24

**Authors:** Kazuyuki Miyashita, Tetsuyuki Yasuda, Hideaki Kaneto, Akio Kuroda, Tetsuhiro Kitamura, Michio Otsuki, Yasuyuki Okamoto, Noboru Hamada, Munehide Matsuhisa, Iichiro Shimomura

**Affiliations:** ^1^Department of Metabolic Medicine, Osaka University Graduate School of Medicine, 2-2 Yamadaoka, Suita, Osaka 565-0871, Japan; ^2^Diabetes Therapeutics and Research Center, The University of Tokushima, Tokushima 770-8503, Japan; ^3^Sumire Clinic, Osaka 535-0031, Japan; ^4^Sumire Hospital, Osaka 536-0001, Japan

## Abstract

We herein report the case of a 41-year-old Japanese female office worker who developed symptomatic hypocalcemia with severe vitamin D deficiency following treatment for Graves' disease with methimazole. The patient's hypocalcemia was mainly caused by vitamin D deficiency due to unbalanced diets and inadequate exposure to sunlight in addition to the resolution of hyperthyroidism. Vitamin D deficiency is increasing worldwide, and it has been more recently shown to relate to the pathogenesis of Graves' disease. However, vitamin D deficiency as a cause of hypocalcemia has received little attention. Taken together, this case suggests that we should take more care in calcium kinetics and vitamin D status during treatment for Graves' disease with antithyroid drugs.

## 1. Introduction

Hypocalcemia following treatment for Graves' disease with thyroidectomy is often caused by postoperative transient hypoparathyroidism and transient increase in bone formation during the resolution of hyperthyroidism [1]. On the other hand, there have been few reports of hypocalcemia following treatment for Graves' disease with antithyroid drugs such as methimazole (MMI) [2]. Calcium metabolism is under control through the coordinated actions of parathyroid hormone (PTH) and activated vitamin D, and the most common cause of hypocalcemia in primary care is vitamin D deficiency [3]. Vitamin D deficiency is increasing worldwide [4–8], and it has been drawing much attention in the association of various diseases including cancer, cardiovascular disease, endocrine and metabolic diseases, and infectious and autoimmune diseases [9–13]. In addition, it has been more recently suggested that vitamin D deficiency is associated with Graves' disease [14–16]. However, vitamin D deficiency as a cause of hypocalcemia has received little attention [17, 18]. In this report, we present the case of a 41-year-old Japanese female office worker who developed symptomatic hypocalcemia with severe vitamin D deficiency following treatment for Graves' disease with MMI. 

## 2. Case Presentation

A 41-year-old Japanese female office worker with Graves' disease was admitted to our hospital for further examination of hypocalcemia in winter. She had no family history of bone and mineral metabolism disorders and never been drunk. She had a previous history of symptomatic gallstones 8 years before admission, since then, she had restricted intake of fat-rich foods such as meats, oily fish, and milk, for preventing abdominal pain due to gallstones. One year before admission, she visited a local hospital with complaints of palpitation, tremor, and weight loss. She was diagnosed as Graves' disease because of goiter and elevated serum thyroid hormones (free triiodothyronine (FT3) 12.96 pg/mL; normal level, 2.0–3.4 pg/mL, free thyroxin (FT4) 3.44 ng/dL; normal level, 0.9–1.6 ng/dL) and undetectable thyroid stimulating hormone (TSH) (<0.01 *μ*U/mL; normal level, 0.4–3.8 *μ*U/mL) with elevated serum TSH receptor antibody (TRAb) (10.7 IU/L; normal level, <1.0 IU/L). At that time, her serum corrected calcium level was normal (8.9 mg/dL; normal level 8.4–10.0 mg/dL). After diagnosis of GD, she was started on treatment with MMI (15 mg once daily) and her hyperthyroidism was gradually corrected. However, about 6 months after treatment with MMI, her thyroid function was severely decreased (FT3 < 0.7 pg/mL, FT4 0.43 ng/dL, TSH 5.81 *μ*U/mL), and she felt numbness in her hands and lip as well as general fatigue. Therefore, MMI was reduced and levothyroxine sodium hydrate (LT4) was started. However, because her numbness was not completely improved after recovery from hypothyroidism, she visited Sumire Clinic for the treatment of her Graves' disease. At that time, she was diagnosed as severe hypocalcemia (serum corrected calcium level, 6.7 mg/dL) and referred to our hospital. On admission, she was 152.3 cm tall and weighed 39.3 kg, with body mass index of 16.9 kg/m^2^. Her blood pressure was 96/58 mmHg, and her pulse rate was 68 beats/min with regular rhythm. Her thyroid gland was swollen diffusely. Her heart and breathing sounds were clear. The abdomen was flat and soft, and liver and spleen were not palpable. Trousseau and Chvostek signs were positive. Chest X-ray was normal. Electrocardiogram revealed a prolonged QTc interval (0.49 sec; normal level, 0.37–0.44 sec). The laboratory findings were shown in Table 1. Blood chemistry showed low serum corrected calcium level (7.3 mg/dL) and phosphate level (2.6 mg/dL; normal level, 2.9–4.8 mg/dL), normal magnesium level (2.4 mg/dL; normal level, 1.8–2.4 mg/dL), and elevated alkaline phosphatase level (389 U/L; normal level, 134–359 U/L). There was no renal and liver dysfunction. Urinary calcium excretion ratio was low (0.37%; normal level, 1-2%). Endocrine examinations showed that serum 25(OH)D level was markedly decreased (<5 ng/mL), and intact PTH (iPTH) and 1,25(OH)_2_D levels were elevated (336.5 pg/mL; normal level 10–60 pg/mL and 76 pg/mL; normal level, 20–60 pg/mL, resp.). Thyroid function was almost normal range under treatments of 5 mg/day of MMI and 50 *μ*g/day of LT4. Markers of bone metabolism showed elevated serum bone alkaline phosphatase level (BAP [56.6 U/L; normal level 13.0–33.9 U/L]) and urinary N-terminal telopeptide of type I collagen level (NTx [100.7 nmolBCE/mmol]). Bone mineral density (BMD) and T-Score in her lumber (L2-4), femoral neck, and distal radius (1/3) regions were all decreased (0.688 g/cm^2^ and −2.9 SD, 0.578 g/cm^2^ and −1.9 SD, and 0.449 g/cm^2^ and −3.4 SD, resp.). From her clinical course and laboratory findings, we diagnosed that her numbness in hands and lip was due to hypocalcemia probably caused by vitamin D deficiency and resolving hyperthyroidism following the treatment with MMI. In addition, we suspected that vitamin D deficiency was mainly due to unbalanced diets restricting fat-rich foods and inadequate exposure to sunlight. Therefore, we started treatment with 3 g/day of calcium lactate hydrate and 0.5 *μ*g/day of 1*α*(OH)D_3_. About 3 months after treatment in spring, cholecystectomy for her gallstones was performed. At that time, her serum 25(OH)D level remained low (7.0 ng/mL) in spite of normalization of serum corrected calcium and iPTH levels (9.2 mg/dL and 52 pg/mL, resp.). After surgery, she completely stopped unbalanced diets restricting fat-rich foods, and the treatment with calcium lactate hydrate and 1*α*(OH)D_3_ was discontinued. However, serum 25(OH)D level (11.0 ng/mL) failed to improve to the normal range, and serum corrected calcium and iPTH levels were lower and upper limits of normal ranges, respectively (8.7 mg/dL and 58 pg/mL, resp.) in spite of euthyroidism under treatment with 5 mg/day of MMI 6 months after surgery. Therefore, we restarted treatment with 0.75 *μ*g/day of 1*α*(OH)D_3_. 

## 3. Discussion

In this report, we presented a case of symptomatic hypocalcemia with severe vitamin D deficiency following treatment for Graves' disease with MMI. Symptomatic hypocalcemia after thyroidectomy for Graves' disease is often experienced in clinical practice [1]. However, to our best knowledge, our case is the first report of symptomatic hypocalcemia with severe vitamin D deficiency following treatment for adult Graves' disease with an antithyroid drug.

It is well known that calcium kinetics and bone metabolisms change during treatment for hyperthyroidism [19]. In the hyperthyroid state, serum calcium level increases because of accelerated bone turnover showing disproportionate increased bone resorption compared to increased bone formation caused by the direct stimulation of bone cells by high thyroid hormone concentrations. On the other hand, serum calcium level declines during the resolution of hyperthyroidism. The underlying cause of decline in serum calcium level is thought to be transient increase in bone formation leading to increase in calcium deposition to bone despite decrease in bone resorption during the resolution of hyperthyroidism [19]. In addition, in patients with thyroidectomy for Graves' disease, postoperative damages of the parathyroid glands can be also causes of hypocalcemia [1]. However, there have been few reports of symptomatic hypocalcemia after treatment with antithyroid drugs for Graves' disease [2]. 

Serum calcium is regulated by the coordinated actions of PTH and activated vitamin D. PTH maintains a positive calcium balance by stimulating calcium resorption in the renal tubular and calcium release from bone. In addition, PTH stimulates renal production of 1,25(OH)_2_D from 25(OH)D through stimulating 1*α*-hydroxylase activity. 1,25(OH)_2_D is the most active form of vitamin D and promotes absorption of calcium from the small intestine and stimulates osteoclast differentiation and calcium reabsorption of bone. Vitamin D is produced mainly through synthesis in the skin by ultraviolet irradiation, with a small contribution from dietary intake [20]. Reduced functions of PTH and/or vitamin D cause hypocalcemia. In the present case, the patient developed hypocalcemia with hypophosphatemia, normal serum creatinine and magnesium, undetectable serum 25(OH)D, and elevated serum iPTH following treatment for Graves' disease with MMI. Therefore, we suspected that the patient's hypocalcemia was mainly caused by vitamin D deficiency in addition to the resolution of hyperthyroidism. 

Vitamin D deficiency is the most common cause of hypocalcemia in primary care [3]. Several conditions cause vitamin D deficiency including insufficient dietary intake, inadequate exposure to sunlight such as housebound status, winter season, high latitudes, dark skin and older age, use of antiepileptic drugs, and malabsorption due to inflammatory bowel disease, gastric surgery, and biliary disease [5, 21–23]. In the present case, the patient restricted intake of fat-rich foods such as meats, oily fish, and milk, for preventing abdominal pain due to gallstones. Therefore, we think that one of the causes of patient's vitamin D deficiency was an insufficient dietary intake. In addition, considering the clinical course that the patient's vitamin D deficiency did not completely recover after stopping unbalanced diets, we suspect that inadequate exposure to sunlight also caused the patient's vitamin D deficiency. In fact, patient's hypocalcemia occurred in winter period, and the patient was an office worker wearing sunscreen cosmetics. The prevalence of vitamin D deficiency (<20 ng/mL) in Japanese general populations in December (winter period) was 26% [4], and wearing a sunscreen with para-aminobenzoic acid reduces vitamin D synthesis in the skin by more than 95% [24]. We also think that the patient already had an asymptomatic mild hypocalcemia after starting unbalanced diets. The development of symptoms of hypocalcemia depends on both the absolute concentration of calcium and the speed of decline in calcium. Therefore, the patient's hypocalcemic symptom such as numbness might not be evident because of mildness and gradual development of hypocalcemia after starting unbalanced diets. However, it became evident because of the dynamic resolution of hyperthyroidism by the treatment with MMI and decrease in ultraviolet irradiation due to seasonal changes. 

Interestingly, in addition to its primary role in bone and mineral homeostasis, it has been shown recently that vitamin D has potent immunomodulatory effects and plays important roles in the pathogenesis of several autoimmune diseases [25]. With regard to Graves' disease, an autoimmune thyroid disease, several studies including our previous studies have shown a role of vitamin D in Graves' disease [14–16, 26–29], and it is suggested that vitamin D deficiency is related to the pathogenesis of Graves' disease [30]. We have recently reported significantly lower levels of 25(OH)D and a greater prevalence of vitamin D deficiency in newly onset Graves' patient compared to those in healthy control [15]. Therefore, we think that vitamin D deficiency in the present case may not be accidental but may be causal concomitance. In addition, taking into account the above reported considerations, we also think that subclinical hypocalcemia in patients with Graves' disease during the resolution of hyperthyroidism with antithyroid drugs may be more than expected because of concomitant vitamin D deficiency. 

In conclusion, we report a case of symptomatic hypocalcemia with severe vitamin D deficiency following treatment for Graves' disease with an antithyroid drug. Because vitamin D deficiency is increasing worldwide, and it not only causes hypocalcemia but also is related to the pathogenesis of Graves' disease, we should take more care in calcium kinetics and vitamin D status during the treatment of Graves' disease with antithyroid drugs. 

## Figures and Tables

**Table 1:** Laboratory data on admission.

WBC	5350	/*μ*L	BUN	4.0	mg/dL	TSH	1.71	*μ*U/mL
RBC	393 × 10^4^	/*μ*L	Cr	0.4	mg/dL	Free T3	1.9	pg/mL
Hb	12.3	g/dL	UA	2.6	mg/dL	Free T4	1.0	ng/dL
Ht	37.5	%	Na	139	mEq/L	Tg-Ab	179.4	IU/mL
Plt	16.9 × 10^4^	/*μ*L	K	4.0	mEq/L	TPO-Ab	63.8	IU/mL
			Cl	105	mEq/L	TRAb	2.7	IU/L
T-Bil	0.6	mg/dL	Ca	7.3	mg/dL			
AST	14	U/L	P	2.6	mg/dL	Intact PTH	336.5	pg/mL
ALT	3	U/L	Mg	2.4	mg/dL	25(OH)D	<5.0	ng/mL
ALP	389	U/L	TG	158	mg/dL	1,25(OH)_2_D	76	pg/mL
*γ*-GTP	9	U/L	HDL-C	48	mg/dL	Calcitonin	15	pg/mL
CPK	75	U/L	LDL-C	129	mg/dL	BAP	56.6	U/L
TP	7	g/dL	Glucose	81	mg/dL	U-NTx	100.7	nmolBCE/mmol
Alb	4.1	g/dL	CRP	<0.04	mg/dL	FECa	0.37	%
